# Prognostic value of circulating tumor DNA in operable non-small cell lung cancer: a systematic review and reconstructed individual patient-data based meta-analysis

**DOI:** 10.1186/s12916-023-03181-2

**Published:** 2023-11-27

**Authors:** Dali Chen, Jinbao Guo, Hao Huang, Lei Tian, Yunbo Xie, Qingchen Wu

**Affiliations:** Department of Cardiothoracic Surgery, the First Affiliated Hospital of Chongqing Medical University, 1# Youyi Road, Yuzhong District, Chongqing, 400016 The People’s Republic of China

**Keywords:** Non-small cell lung cancer, Circulating tumor DNA, Disease-free survival, Meta-analysis, Individual patient data

## Abstract

**Background:**

This reconstructed individual patient data (IPD)-based meta-analysis is aimed to summarize the current findings and comprehensively investigate the predictive value of circulating tumor DNA (ctDNA) in operable non-small cell lung cancer (NSCLC).

**Methods:**

PubMed, Cochrane and Embase were searched to include potentially eligible studies. The primary outcomes included progression-free survival (DFS) by ctDNA status at baseline, postoperative, and longitudinal timepoints. The IPD-based survival data was retracted and used in reconstructed IPD-based meta-analysis. Subgroup analysis was implemented based on the baseline characteristics.

**Results:**

Totally, 28 studies were involved, including 15 full-length articles (1686 patients) for IPD-based synthesis and 20 studies for conventional meta-analysis. The IPD-based meta-analysis discovered that patients with positive ctDNA status at the baseline (hazard ratio, HR = 3.73, 95% confidential interval, CI: 2.95–4.72), postoperative (3.96, 2.19–7.16), or longitudinal timepoints (12.33, 8.72–17.43) showed significantly higher risk of recurrence. Patients with persistent ctDNA-negative status had the lowest recurrence rate, and the negative conversion of ctDNA from baseline to postoperative timepoints was correlated with elevated DFS. Subgroup analyses suggested that stage II–III patients with ctDNA-positive status may achieve preferable therapeutic outcomes.

**Conclusions:**

Plasm ctDNA monitoring shows excellent clinical significance at the tested timepoints. Perioperative conversion of ctDNA status may indicate the therapeutic effect of radical surgery. Postoperative adjuvant therapy may be determined according to the ctDNA status.

**Trail registration:**

CRD42022304445.

**Supplementary Information:**

The online version contains supplementary material available at 10.1186/s12916-023-03181-2.

## Background

Lung cancers still are the severest health hazard to people worldwide compared to other types of carcinomas [1, 2]. Non-small cell lung cancers (NSCLC) contribute to most of the subtypes of lung cancers. With the advancement of physical examination, diagnosis, and treatment methods, more and more NSCLC patients can be treated at early stages. The survival outcomes can be significantly improved, and some patients may even be cured [3]. Unfortunately, a part of patients facing disease progression and poor prognosis are still recognized at early stages by the current tumor, node, metastasis (TNM) staging system. Hence, the potential imperfections of the current staging system need to be made up by involving effective molecular biomarkers.

Circulating tumor DNA (ctDNA), which is defined as a specific portion of cell-free DNA comprising strands of < 145 bp in length, is recognized as a preferable biomarker of disease progression for various cancers, including NSCLC [4–6]. The predictive role of ctDNA was initially investigated at advanced stages of NSCLC, which shows ctDNA has excellent value in predicting disease progression. A multi-center cohort study by prospectively including 1127 advanced NSCLC patients suggests that ctDNA detection in plasma is an independent predictor of survival (hazard ratio (HR), 2.05; 95% confidence interval (CI), 1.74–2.42) [7].

Researchers doubt whether or not ctDNA can make a difference in patients at early stages of NSCLC [8]. Recently, this question has been investigated. Some publications with comparatively small sample sizes fail to identify the prognostic role of ctDNA [9–11]. Nevertheless, most of the studies present positive results. After analyzing the clinical data of 116 participants, Qiu B et al. discovered that patients with postoperative and longitudinal negative ctDNA had significantly better recurrence-free survival [12]. Zhang JT et al. confirmed the clinical significance of ctDNA by including a comparatively large sample size [13]. They further imply that patients with negative ctDNA status may not benefit from postoperative adjuvant treatment.

We have discovered several disadvantages after reviewing these references, which need to be reinforced. A majority of these studies include relatively small sample sizes, and many studies recruit less than 50 patients. The baseline characteristics are unbalanced between groups, which has not been dealt with using statistical methods. In addition, there is a lack of subgroup analysis. Therefore, our reconstructed individual patient data (IPD) meta-analysis trys to summarize the existing findings, comprehensively investigate the predictive value of ctDNA in early stage (I–III) NSCLC and make a guidance for future studies.

## Methods

According to the Preferred Reporting Items for Systematic Reviews and Meta-Analyses (PRISMA) IPD checklist (Additional file 1), we conducted this reconstructed IPD-based meta-analysis, which was registered aforehand with the International Prospective Register of Systematic Reviews (PROSPERO) (CRD42022304445).

### Criteria of eligible studies and individual participants

According to the NCCN guideline, we defined the operable NSCLC as patients with I–III stage who underwent radical surgery. Prospective/retrospective cohort or case–control studies were preferred for inclusion, because randomized clinical trials were impracticable for exploring the prognostic role of ctDNA in NSCLC. Full-length articles and abstract studies focusing on the study topic were all eligible. Studies with specific survival data were included in reconstructed IPD-based meta-analysis and conventional meta-analysis, and otherwise, they were included in systematic review. Stage I–III NSCLC patients with radical surgery were eligible for the IPD-based meta-analysis. Patients without survival data were excluded. Patients with R-1/R-2 resection were further excluded from the synthesis. No restriction was set in terms of ctDNA testing methods and definitions of ctDNA positive status.

### Literature retrieval

Three major online databases (PubMed, Embase, and Cochrane) were searched for potentially eligible studies. The detailed search strategies were displayed in Additional file 2. The last retrieval date was April 22, 2022, without language limitation. We manually searched the reference lists of the full-length articles so as to avoid omission. At last, search in the three databases was updated before data analysis to discover newly published studies that may conform to the inclusion criteria.

### Study selection and assessment

Two researchers independently completed the entire screen process. Any disagreement between them was solved by discussing with a third researcher. The potential literature was gathered and screened on EndNote X7 (Clarivate Analytics Co. Ltd.), and a PRISMA flow diagram was reported. Three steps of selection were taken. First, the researchers excluded duplications. Then, they screened the rest of studies using titles and abstracts to determine which studies will be included for further filtration. Finally, the full texts of articles were screened, and eligible studies were included for meta-analysis. The two independent researchers assessed the quality of the studies included in quantitative synthesis using the Newcastle–Ottawa Scale (NOS). The NOS contains three parts, including selection, comparability, and outcome, and studies were scored from 0 to 9.

### Data extraction

The two researchers independently extracted the targeted data, and any discrepancy between them was solved by discussing with a third researcher. The data were extracted in studies involved in the quantitative analysis and mainly contained two parts: baseline characteristics and survival outcomes. The baseline characteristics included study type, neoadjuvant therapy (NAT), ctDNA panel, testing timepoints, definition of ctDNA-positive, postoperative adjuvant chemotherapy (ACT), location, sample size, pathological stage, median follow-up period, and precedence of relapse. In our subsequent analysis, patients receiving other types of postoperative treatment were also included in the ACT subgroup, such as adjuvant radiotherapy, adjuvant chemoradiotherapy, target therapy, and immune checkpoint inhibitors (ICIs). In terms of survival outcomes, we firstly collected the reported HRs and 95%CI from the included articles and abstracts. The IPD-based survival data was retracted from the published supplemental files of all included full-length articles. Otherwise, the reported Kaplan–Meier curves and swimmer plots were used to collect corresponding survival and ctDNA status data using the methods reported by Guyot et al. [14]. The demographic variables of individual patients were also collected, such as age, gender, smoking history, tumor size (cm), pathological type, pathological stage (T&N), ACT, NAT, and ctDNA status at different timepoints. Longitudinal ctDNA positive status was defined as ctDNA positive at any one testing timepoint during follow-up period.

### Statistical analysis

We focused on the prognostic role of ctDNA in operable NSCLC. The primary outcome was IPD-based disease-free survival (DFS) by ctDNA status at baseline, postoperative, and longitudinal timepoints. The secondary outcomes included overall survival (OS) and the predictive value of ctDNA on recurrence. The survival data of ctDNA status at various testing timepoints was collected and analyzed. In IPD analysis, survival curves were plotted using the Kaplan–Meier method, and the HRs (95% CI) of DFS/OS were calculated based on the Cox proportional hazards model. Sensitivity analyses were conducted to address the between-study heterogeneity using stratified Cox regression and shared frailty models. Multivariate Cox regression was implemented to balance inter-group differences of baseline characteristics, including age, gender, smoke, tumor size, pathological type, T stage, N stage, and pathological stage. Propensity score matching (PSM) was also used to flush discrepancy of baseline characteristics between ctDNA-positive and ctDNA-negative groups, which allowed to calculate HRs (95% CI) based on similar between-group demographic variables. Five covariates (age, gender, smoking history, pathological type, and stage) were included in PSM. The nearest neighbor method was used to match (ratio = 1:1) patients between groups with no replacement. The caliper width was set at 0.02. Furthermore, subgroup analysis was conducted for age, gender, smoking history, tumor size, pathological types, T stage, N stage, pathological stage, and ACT.

In conventional meta-analysis, we synthesized the reported HRs (95% CI) from the included studies based on a fixed/random-effect model, which was chosen depending on the level of inter-study heterogeneity. We defined the significance of heterogeneity as *I*^2^ > 50% or *P* < 0.05, and a random-effect model was implemented. Sensitivity analysis was conducted to discover potential sources of inter-study heterogeneity. Funnel plots were used to assess potential publication bias.

The statistical significance was defined as two-side *P* < 0.05. The analyses were conducted on RStudio 4.0.2 (R Project for Statistical Computing) and SPSS 23.0 (SPSS Inc., Chicago, IL, USA).

## Results

Online database search identified 2487 articles, of which 1638 records were retrieved after removing duplicates (Figure S1). Finally, 28 studies were included, including 15 full-length articles and 13 abstract studies. Among them, 7 abstract studies without specified survival data were included in the systematic review, [15–21]. 15 full-length articles were included in the IPD-based synthesis, [10–13, 22–32] and 20 studies with detailed survival data were included in the conventional synthesis of HRs (95% CI) [9–13, 23–37]. A full-length study by Zhao X et al. [22], which reported survival outcomes of 7 patients without HR (95% CI), was only included in the IPD-based synthesis. All studies included in quantitative analysis were assessed as high-quality studies (scored no less than 7 points) based on the NOS (Table S1).

The detailed baseline characteristics and survival outcomes of the included studies for meta-analysis were summarized in Tables S2 and S3, respectively. The 21 studies totally involved 1686 participants, ranging from 7 to 330 patients in each study. The first three testing timepoints were baseline, postoperative, and longitudinal timepoints (Figure S2). Additional file 3 summarized the IPD of 15 studies, the data from three of which was reconstructed from the reported Kaplan–Meier (K-M) curves [13, 23, 26]. Half of the included participants were younger than 60 years. Most of the patients (70.65%) were diagnosed as adenocarcinoma (AD) in the mean tumor size of 2.79 ± 1.41 cm. The detailed baseline characteristics of IPD were summarized in Table S4. In addition, inter-group disparities of baseline characteristics at the baseline, postoperative, and longitudinal timepoints were almost balanced using PSM (Figure S3; Tables S5 to S7 respectively).

### Prognostic role of baseline ctDNA status

**Fig. 1:**
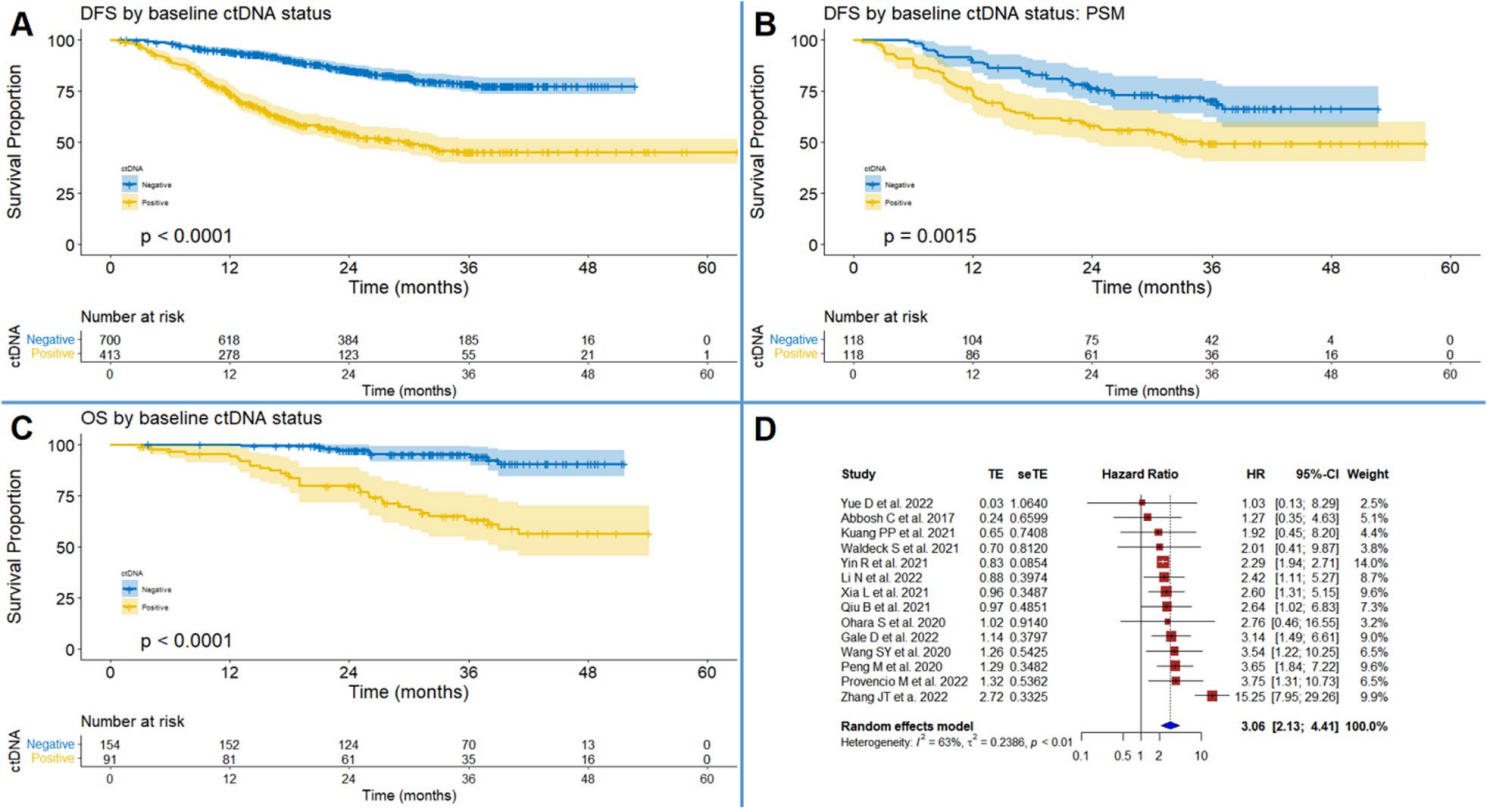
Synthesis of survival outcomes by baseline ctDNA status. **A** Individual patient data (IPD)-based disease-free survival (DFS) by baseline ctDNA status (+ vs. −). **B** IPD-based DFS by baseline ctDNA status (+ vs. −) based on propensity score matching (PSM). **C** IPD-based overall survival (OS) by baseline ctDNA status (+ vs. −). **D** Pooled meta-analysis of DFS by baseline ctDNA status (+ vs. −)

Totally, 13 studies [10–13, 22, 24–31] involving 1113 participants were included in the IPD-based DFS analysis (Fig. 1A). Patients with negative ctDNA status showed significantly lower recurrence rate than those with positive ctDNA status (HR = 3.73, 95% CI: 2.95–4.72, *P* < 0.001). Totally, 118 pairs of participants from 5 studies [24, 25, 27, 29, 31] were generated with PSM. The survival analysis indicated that the difference in recurrence rate between patients with different ctDNA statuses remained significant (HR = 1.97, 95% CI: 1.29–3.01, *P* = 0.002, Fig. 1B). Another analysis via multivariate Cox regression with 610 patients from 5 studies [24, 25, 27, 29, 31] generated similar estimate (*P* < 0.001, Table S8). Furthermore, sensitivity analyses by Cox regression with a shared frailty model and a stratified Cox regression model, which modeled between-study heterogeneity, confirmed the consistency of estimates (Table S8). IPD-based OS analysis based on 245 patients from 4 studies [22, 26, 27, 29] also yielded HRs in favor of ctDNA-negative patients (Fig. 1C). The results of subgroup analysis were summarized in Table S8. In addition, the pooled HRs (95% CI) of DFS and OS by conventional meta-analysis showed favorable prognostic value of ctDNA negative status (Fig. 1D and Figure S4A, respectively). Sensitivity analysis indicated that after removing the study by Zhang JT et al. [13], the between-study heterogeneity was reduced significantly (Figure S4B) and no publication bias was discovered (Figure S4C).

### Prognostic role of postoperative ctDNA status

**Fig. 2:**
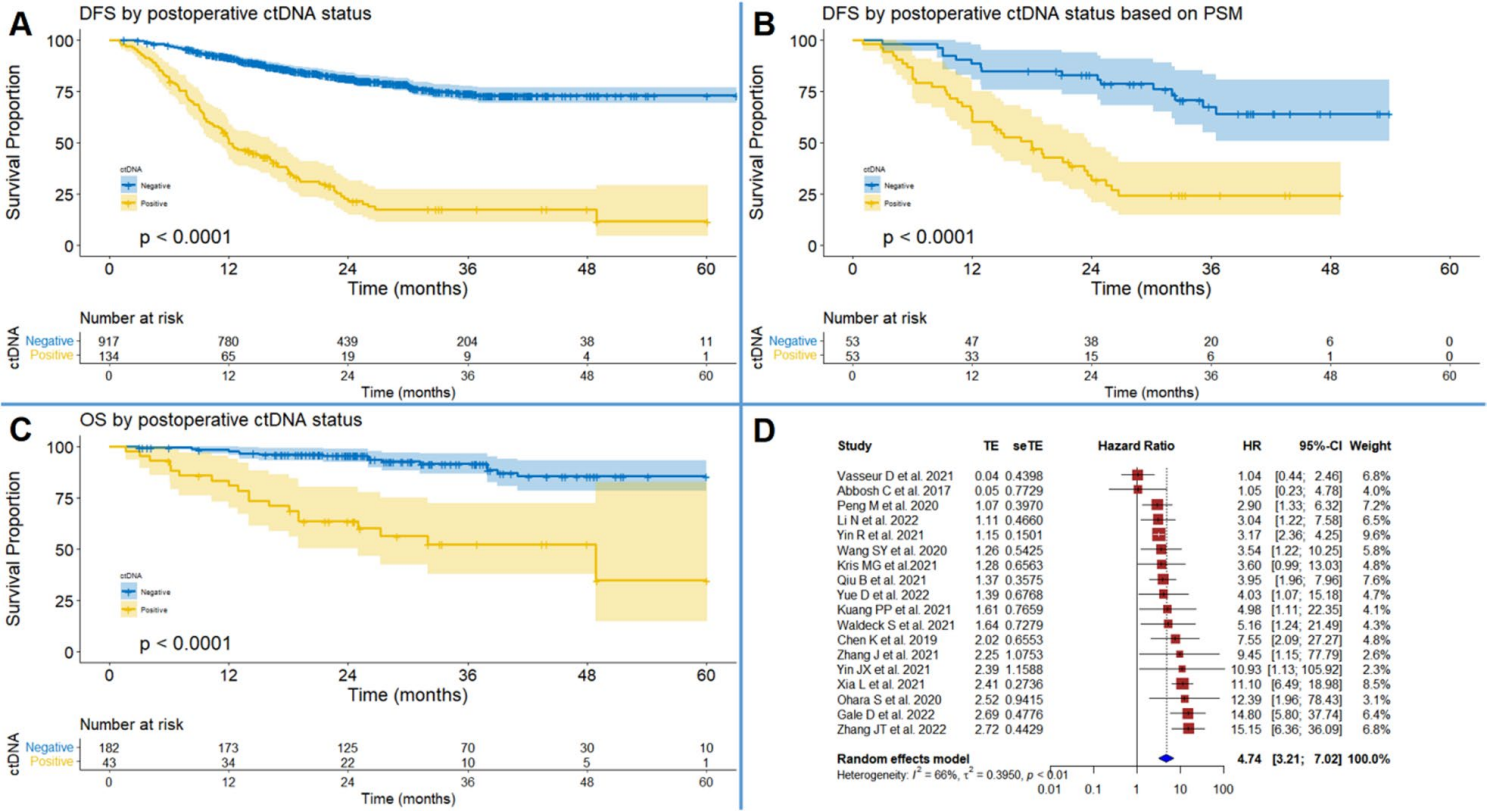
Synthesis of survival outcomes by postoperative ctDNA status. **A** Individual patient data (IPD)-based disease-free survival (DFS) by postoperative ctDNA status (+ vs. −). **B** IPD-based DFS by postoperative ctDNA status (+ vs. −) based on propensity score matching (PSM). **C** IPD-based overall survival (OS) by postoperative ctDNA status (+ vs. −). **D** pooled meta-analysis of DFS by postoperative ctDNA status (+ vs. −)

Totally, 1051 participants from 14 studies [10–13, 22–25, 27–32] were included in the IPD-based DFS analysis (Fig. 2A). Postoperative negative ctDNA status predicted significantly lower recurrence rate than positive ctDNA status (HR = 6.52, 95% CI: 5.08–8.36, *P* < 0.001). PSM generated 53 pairs of participants from 5 studies [24, 25, 27, 29, 31], and the survival analysis confirmed the correlation of postoperative ctDNA positive status with higher risk of recurrence (HR = 3.96, 95% CI: 2.19–7.16, *P* < 0.001, Fig. 2B). Inter-group demographic discrepancy was also addressed using multivariate Cox regression analysis, which showed similar estimate (*P* < 0.001, Table 1). Furthermore, sensitivity analyses by Cox regression with the shared frailty model and the stratified Cox regression model confirmed the consistency of estimates (Table 1). IPD-based OS analysis with 225 patients from 5 studies [22, 23, 27, 29, 32] also yielded HRs in favor of ctDNA-negative patients (Fig. 2C). The results of subgroup analysis were summarized in Table 1. In addition, the pooled HRs (95% CI) of DFS and OS by conventional meta-analysis showed favorable prognostic value of postoperative ctDNA negative status (Fig. 2D and Figure S4D, respectively). Sensitivity analysis failed to identify the source of between-study heterogeneity (Figure S4E) and the funnel plot showed no publication bias (Figure S4F).

**Table 1:** Survival outcomes by postoperative ctDNA status (+ vs −) based on reconstructed individual patient data

Group (cases/ref.)	Subgroup (cases/ref.)	DFS/OS	HR	LCI	UCI	*P* value
Postoperative ctDNA (225/5)		OS	6.44	3.31	12.54	< 0.001
Postoperative ctDNA (225/5)^a^		OS	5.59	2.84	10.98	< 0.001
Postoperative ctDNA (225/5)^b^		OS	5.08	2.58	9.99	< 0.001
Postoperative ctDNA (1051/14)		PFS	6.52	5.08	8.36	< 0.001
Postoperative ctDNA (1051/14)^a^		DFS	5.88	4.51	7.67	< 0.001
Postoperative ctDNA (1051/14)^b^		DFS	5.85	4.46	7.67	< 0.001
Postoperative ctDNA (538/5)^c^		DFS	3.87	2.57	5.83	< 0.001
Postoperative ctDNA by PSM (106/5)		DFS	3.96	2.19	7.16	< 0.001
Age	< 60 years (270/6)	DFS	8.01	4.48	14.32	< 0.001
	60–69 years (194/5)	DFS	5.28	2.97	9.37	< 0.001
	70–75 years (69/6)	DFS	7.53	2.57	22.04	0.001
	> 75 years (45/5)	DFS	2.40	0.94	6.18	0.090
Gender	Female (260/6)	DFS	6.44	3.30	12.57	< 0.001
	Male (318/6)	DFS	5.52	3.61	8.44	< 0.001
Smoking history	With (299/6)	DFS	5.66	3.47	9.24	< 0.001
	Without (358/5)	DFS	5.62	3.69	8.55	< 0.001
Tumor size	≤ 2 cm (188/3)	DFS	8.40	3.21	21.95	< 0.001
	2.1–3 cm (157/4)	DFS	7.66	3.87	15.15	< 0.001
	3.1–5 cm (133/4)	DFS	4.98	2.63	9.44	< 0.001
	> 5 cm (35/4)	DFS	1.11	0.32	3.82	0.913
Pathological type	AD (534/10)	DFS	6.93	4.73	10.17	< 0.001
	SCC (161/10)	DFS	3.56	2.06	6.15	< 0.001
	Other (43/8)	DFS	3.04	1.26	7.36	0.022
T stage	T1 (298/7)	DFS	8.56	4.58	15.99	< 0.001
	T2 (235/7)	DFS	4.74	2.98	7.53	< 0.001
	T3&T4 (87/7)	DFS	2.16	1.09	4.28	0.043
N stage	N0 (419/8)	DFS	4.82	2.72	8.54	< 0.001
	N1 (97/7)	DFS	4.69	2.45	8.96	< 0.001
	N2 (114/8)	DFS	3.62	2.10	6.25	< 0.001
Pathological stage	Stage I (376/9)	DFS	5.50	2.77	10.92	< 0.001
	Stage II (160/9)	DFS	4.83	2.68	8.68	< 0.001
	Stage III (153/9)	DFS	2.80	1.79	4.37	< 0.001
ACT	With (310/10)	DFS	4.06	2.70	6.09	< 0.001
	Without (341/8)	DFS	10.48	6.65	16.52	< 0.001

*ACT* adjuvant chemotherapy, *AD* adenocarcinoma, *HR* hazard ratio, *LCI* lower level of 95% confidential interval, *Other* other types of NSCLC, *OS* overall survival, *DFS* disease-free survival, *PSM* propensity score matching, *SCC* squamous cell carcinoma, *UCI* upper level of 95% confidential interval

^a^The HR (95% CI) was calculated based on Cox regression with shared frailty model

^b^The HR (95% CI) was calculated based on a stratified Cox regression model

^c^The HR (95% CI) was calculated based on a multivariate Cox regression model including age, gender, smoke, tumor size, pathological type, T stage, N stage, and pathological stage

### Prognostic role of longitudinal ctDNA status

**Fig. 3:**
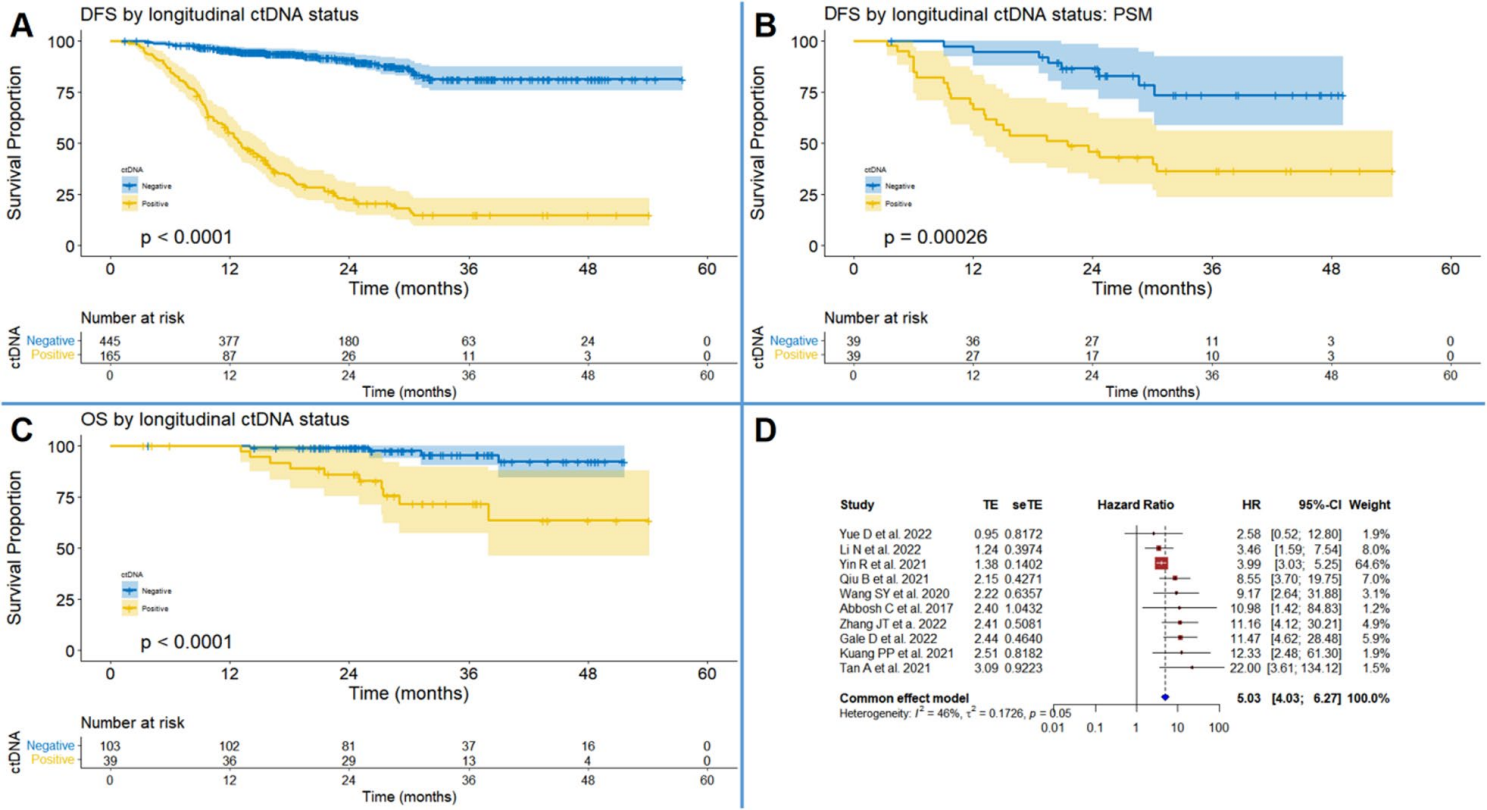
Synthesis of survival outcomes by longitudinal ctDNA status. **A** Individual patient data (IPD)-based disease-free survival (DFS) by longitudinal ctDNA status (+ vs. −). **B** IPD-based DFS by longitudinal ctDNA status (+ vs. −) based on propensity score matching (PSM). **C** IPD-based overall survival (OS) by longitudinal ctDNA status (+ vs. −). **D** pooled meta-analysis of DFS by longitudinal ctDNA status (+ vs. −)

A total of 8 studies [10–13, 27, 29–31] involving 610 participants were included in the IPD-based DFS analysis (Fig. 3A). During the follow-up period, patients with persistent negative ctDNA showed significantly lower recurrence rate than those with positive ctDNA (HR = 12.33, 95% CI: 8.72–17.43, *P* < 0.001). Totally, 39 pairs of participants from 3 studies [27, 29, 31] were generated with PSM, and the survival analysis showed that the between-group difference of recurrence rate remained significant (HR = 3.99, 95% CI: 1.76–8.89, *P* < 0.001, Fig. 3B). Another method via multivariate Cox regression helped to address inter-group demographic discrepancy, which generated similar estimate (*P* < 0.001, Table S9). Sensitivity analyses by Cox regression with the shared frailty model and the stratified Cox regression model confirmed the consistency of estimates (Table S9). IPD-based OS analysis with 142 patients from 2 studies [27, 29] also yielded HRs in favor of patients with longitudinal ctDNA negative status (Fig. 3C). The results of subgroup analysis were summarized in Table S9. In addition, the pooled HRs (95% CI) of DFS by conventional meta-analysis showed favorable prognostic value of ctDNA negative status (Fig. 3D). Sensitivity analysis showed that the between-study heterogeneity mainly originated from the study by Yin R et al. [34] (Figure S4G), and visional publication bias was discovered (Figure S4H).

### Prognostic roles of ctDNA status after NAT/ACT

Totally, 62 patients from 2 studies [11, 26] were included to evaluate the IPD-based prognostic role of post-NAT ctDNA status (Figure S5A). Negative ctDNA status predicted significantly lower recurrence incidence than positive ctDNA status (HR = 4.55, 95% CI: 1.72–12.02, *P* = 0.002). The synthesis of HRs (95% CI) from the two studies showed a similar result (Figure S5B). Another 2 studies [12, 30] involving 114 participants reported the DFS outcomes according to different ctDNA statuses after ACT. The pooled IPD-based analysis insisted that positive ctDNA status after ACT was associated with significantly higher risk of recurrence (HR = 3.96, 95% CI: 2.08–7.52, *P* < 0.001, Figure S5C). The pooled estimate of the reported HRs (95% CI) confirmed the significant correlation between positive ctDNA status after ACT and higher recurrence risk (Figure S5D).

### Predictive values of ctDNA at various timepoints

We further reported the pooled positive predicted value (PPV) and negative predictive value (NPV) for different ctDNA statuses at various testing timepoints through subgroup analysis (Table S10). In all, the NPV of ctDNA was higher than its PPV at all the three timepoints. Individually, the lowest PPV was reported at the baseline timepoint (43.94%). Longitudinally, the combined predictive value (CPV) of ctDNA gradually increased from 63.72% at the baseline timepoint to 83.48% at the longitudinal timepoint. From the subgroup perspective, the gap between NPV and PPV shrank with the elevation of pathological stage (Table S10). In the stage I subgroup, the ctDNA status was more clinically significant in terms of NPV versus PPV at any timepoint. Patients with AD showed comparable CPV versus those with squamous cell carcinoma (SCC) at all timepoints except for the baseline timepoint (68.20% vs. 58.40%). Entirely, ctDNA testing displayed the best clinical predictive value at the longitudinal follow-up period.

### ctDNA status change and ACT decision-making

**Fig. 4:**
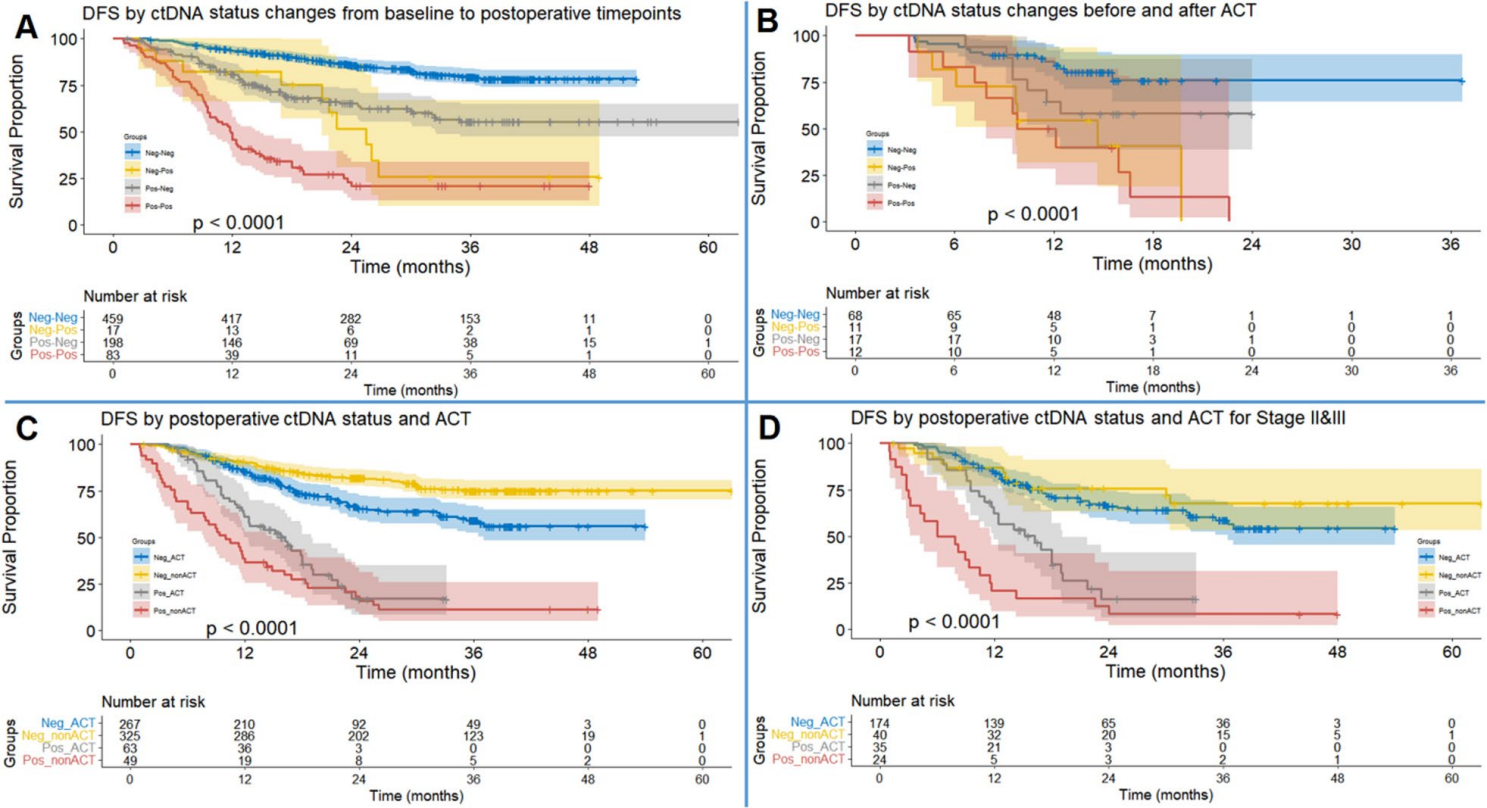
Synthesis of individual patient data (IPD)-based disease-free survival (DFS) by combined analysis of two parameters. **A** DFS by ctDNA status change from baseline to postoperative timepoints. **B** DFS by ctDNA status change before and after ACT. **C** DFS by postoperative ctDNA status and whether adjuvant chemotherapy (ACT) was conducted or not. **D** DFS by postoperative ctDNA status and whether ACT was conducted or not for patients at stage II&III

The change in the prognostic role of ctDNA status from baseline to postoperative timepoint was evaluated by including 757 participants from 11 studies [10–12, 22, 24, 25, 27–31] (Fig. 4A). Persistent ctDNA negative status predicted the lowest recurrence rate, and continuous ctDNA positive status was correlated with the poorest result of DFS (Table 2). Subgroup analyses by pathological types and stages were summarized in Table S11 (Figure S6). Furthermore, the relationship between ctDNA status change after ACT and recurrence risk was also explored by involving 108 patients from 3 studies [12, 13, 30] (Table 2). Patients with persistent ctDNA negative status showed the best DFS outcome, and patients with persistent ctDNA positive status showed the highest risk of recurrence (Fig. 4B).

**Table 2:** Survival outcomes by three combinations of two parameters: ctDNA status change from baseline to postoperative timepoints, postoperative ctDNA status and whether ACT was conducted or not, and ctDNA status change before and after ACT

Subgroup	HR	LCI	UCI	*P* value	HR	LCI	UCI	*P* value	HR	LCI	UCI	*P* value
**DFS by ctDNA status changes from baseline to postoperative timepoints**												
Neg-Neg	1.00 (ref.)											
Neg-Pos	4.59	2.37	8.87	< 0.001	1.00 (ref.)							
Pos-Neg	2.79	2.02	3.87	< 0.001	0.60	0.31	1.16	0.126	1.00 (ref.)			
Pos-Pos	9.01	6.41	12.72	< 0.001	1.92	0.98	3.75	0.058	3.16	2.22	4.50	< 0.001
**DFS by postoperative ctDNA status and ACT**												
Neg_ACT	1.00 (ref.)											
Neg_nonACT	0.54	0.39	0.74	< 0.001	1.00 (ref.)							
Pos_ACT	3.16	2.17	4.60	< 0.001	5.70	3.83	8.50	< 0.001	1.00 (ref.)			
Pos_nonACT	4.79	3.31	6.94	< 0.001	8.69	5.89	12.83	< 0.001	1.50	0.97	2.32	0.067
**DFS by postoperative ctDNA status and ACT for stage II/III NSCLC**												
Neg_ACT	1.00 (ref.)											
Neg_nonACT	0.76	0.40	1.45	0.401	1.00 (ref.)							
Pos_ACT	2.96	1.85	4.76	< 0.001	3.62	1.76	7.48	< 0.001	1.00 (ref.)			
Pos_nonACT	6.59	4.02	10.80	< 0.001	7.99	3.83	16.68	< 0.001	2.21	1.23	3.95	0.008
**DFS by ctDNA status changes before and after ACT**												
Neg-Neg	1.00 (ref.)											
Neg-Pos	4.46	1.75	11.35	0.002	1.00 (ref.)							
Pos-Neg	2.06	0.80	5.27	0.132	0.45	0.16	1.30	0.452	1.00 (ref.)			
Pos-Pos	5.49	2.35	12.80	< 0.001	1.21	0.46	3.22	0.701	2.67	1.01	7.02	0.047

*ACT* (with) adjuvant chemotherapy, *HR* hazard ratio, *LCI* lower 95% confidential interval, *nonACT* without adjuvant chemotherapy, *NSCLC* non-small cell lung cancer, *DFS* disease-free survival, *Pos* ctDNA positive, *Neg* ctDNA negative, *UCI* upper 95% confidential interval

We also explored the prognostic role of ACT in patients with different postoperative ctDNA statuses. Eleven studies [10, 12, 13, 22, 24, 25, 27, 28, 30–32] involving 704 participants were included. In the postoperative ctDNA negative group, patients without ACT showed significantly superior DFS outcome than those with ACT (HR = 0.54, 95% CI: 0.39–0.74, *P* < 0.001, Fig. 4C). In the postoperative ctDNA positive group, ACT can reduce the recurrence rate, though not significantly (*P* = 0.067, Table 2). After excluding patients at stage I, we discovered that ACT significantly improved the DFS outcome of patients with postoperative ctDNA positive status (HR = 2.21, 95% CI: 1.23–3.95, *P* = 0.008, Fig. 4D). However, ACT did not improve the survival of patients with postoperative ctDNA negative status (*P* = 0.401, Table 2). Furthermore, ACT was associated with poor survival outcomes among ctDNA- negative patients in subgroups of stage I and AD (Table S12). ACT failed to differentiate patients with ctDNA positive status in stages I and SCC (Figure S7). Therefore, these results imply that ACT may obtain preferable therapeutic outcomes in stage II–III and the AD subgroup for ctDNA-positive patients.

## Discussion

To our knowledge, this is the first reconstructed IPD-based meta-analysis to comprehensively investigate the prognostic role of ctDNA in patients with stage I–III NSCLC. Patients with positive ctDNA status have significantly poorer prognosis (both DFS and OS) than those with negative ctDNA status. The prognostic value of monitoring ctDNA status is increasing with time from baseline to longitudinal timepoints, which is further confirmed by the analysis of clinical predictive values (PPV&NPV). We also conducted subgroup analyses at baseline, postoperative, and longitudinal timepoints, which show the estimates are consistent in almost all subgroups. In addition, the role of ctDNA status in determining whether ACT shall be conducted or not was confirmed by our analysis. We discovered that only patients with postoperative ctDNA positive status may benefit from ACT. Subgroup analyses further identified that the beneficial effects mainly helped stage II–III and AD subgroups.

The quality of each included study was assessed as high level according to the NOS. Most of the studies were designed as prospective cohort studies. The definitions of critical variables were clearly described in the method part of the published articles, such as ctDNA panel formation, definition of ctDNA positive status, and testing timepoints. Some studies [13, 24] described the postoperative and longitudinal ctDNA positive statuses as molecular residual disease (MRD), which was not used in other included studies. A part of patients with postoperative ctDNA positive status still survived without recurrence. Thus, we did not describe the postoperative or longitudinal ctDNA positive status as MRD. The major defect of the existing study designs is between-group comparability. Only several studies with comparatively large sample sizes reported sensitivity analysis by accounting for the heterogeneity of between-group baseline characteristics [12, 13]. We speculate that small sample size is the major cause for the imperfection. Therefore, to cover the shortage, we used reconstructed IPD-based synthesis with several types of sensitivity analyses (e.g., multivariate Cox regression and PSM) to handle demographic disparities between groups. In addition, inter-study heterogeneity was further dealt with using sensitivity analysis based on stratified Cox regression and shared frailty models.

The existing studies focus on the clinical value of plasm ctDNA monitoring at three timepoints, including baseline, postoperative, and longitudinal timepoints. In our study, the baseline timepoint was defined as the time after diagnosis and before any treatment, including NAT and radical surgery. Reportedly, postoperative ctDNA positive status within 3 days rapidly degraded after radical surgery and showed no prognostic value of recurrence [32]. Thus, the postoperative timepoint was defined as the time duration from 3 days to 1 months after surgery. The longitudinal timepoint was defined as any timepoint during the follow-up period (once per 3–6 months). Some studies suggest that ctDNA at baseline timepoint is not an independent predictor of recurrence. A study with 20 participants implies that the presence of ctDNA at diagnosis is not associated with recurrence-free survival in the follow-up with a median duration of 30 months [19]. However, after analyzing preoperative plasma samples of 55 early-stage NSCLC patients, Cho JH et al. [21] reported an opposite outcome in terms of the prognostic role of baseline ctDNA status. Our synthetic findings demonstrate that baseline positive ctDNA status can be an independent predictor of prognosis (both recurrence and OS) for operable NSCLC (stage I–III).

In terms of postoperative ctDNA status, Vasseur D et al. [9] failed to identify its relationship with disease-free survival or OS. Simon N et al. [18] investigated 70 samples and insisted that postoperative plasma ctDNA may be a potential vicious biomarker of recurrence. A small pilot cohort showed that postoperative ctDNA positive status was significantly associated with recurrence, although the absence of plasma ctDNA was not associated with the lack of recurrence [20]. In all, the majority of the included studies demonstrate that postoperative ctDNA positive status is significantly correlated with higher risk of recurrence [15, 16]. Our study confirms that the postoperative presence of ctDNA can be an independent prognostic biomarker for operable NSCLC (stages I–III), which shows better predictive value than the baseline ctDNA status. In terms of longitudinal ctDNA monitoring, all studies discover that persistent absence of plasma ctDNA during the follow-up period predicts a significantly higher probability of curing, which is also verified by our analysis. Moreover, the clinical predictive value of plasma ctDNA monitoring peaks at the longitudinal timepoint compared with the baseline and postoperative timepoints.

The existing studies did not investigate the impact of baseline factors on the prognostic value of ctDNA status. Our analyses show that the prognostic value of ctDNA status exists in almost all subgroups based on baseline characteristics. The prognostic value of ctDNA is apparently higher at stage I than at stage II/III and is lower in the ACT group than in the non-ACT group. Furthermore, we evaluated the correlation between ctDNA status change after radical surgery and DFS. Results demonstrate that patients with persistent ctDNA negative status have better survival outcomes than patients with ctDNA negative conversion and show an elevated DFS versus those with ctDNA positive conversion or continuous ctDNA positive status. Similar results were identified in the group of ctDNA status change after ACT. Additionally, we investigated the HRs (95% CI) of DFS by combining postoperative ctDNA status and ACT and found only stage II–III patients with postoperative ctDNA positive status benefited from ACT. Subgroup analysis by pathological types further showed the improved DFS due to ACT mainly came from the AD group rather than the non-AD group.

Several limitations exist in our study. Firstly, we failed to collect the survival data of some included studies and reconstructed these data using reported statistical methods. Even though the reported K-M curves can be reproduced using our reconstructed survival data, this may impair the final results of our study. Secondly, we failed to retract the baseline characteristics of patients in some studies and thereby had to exclude these patients from multivariate Cox regression analysis and PSM. The quality of included abstract studies failed to be comprehensively reviewed. It might bring about some bias in our meta-analysis. Thirdly, we assigned several types of postoperative adjuvant therapy into the ACT group, including ACT, radiotherapy, and chemoradiotherapy. Unfortunately, targeted therapy and ICIs only occupied a very tinny proportion. Therefore, our results about the clinical value of ctDNA for ACT are inapplicable to targeted therapy and ICI treatment. Furthermore, because of the variation of ctDNA panels among included studies, the definition of ctDNA positive status varied among these included studies. We failed to unify the definitions of ctDNA positive status, and the difference of ctDNA panels between the included studies may lead to bias in our results. Additionally, most of the included studies came from mainland China, which may limit the application of our conclusions into other ethnic populations. Thus, the prognostic role of ctDNA in operable NSCLC shall be further investigated in patients of other races.

## Conclusions

Several progressive findings were obtained. Our IPD-based meta-analysis suggests that plasma ctDNA monitoring is very clinically significant at all tested timepoints, as confirmed by subgroup analyses. The prognostic value of ctDNA status monitoring is increasing from baseline to longitudinal timepoints. The perioperative conversion of ctDNA status may indicate the therapeutic effect of radical surgery. The postoperative adjuvant therapy may be determined according to the ctDNA status. The treatment effects of ACT mainly benefit stage II-III patients with postoperative ctDNA positive status.

### Supplementary Information

**Additional file 1.** Preferred Reporting Items for Systematic Reviews and Meta-Analyses (PRISMA) checklist for individual patient data-based meta-analysis.

**Additional file 2.** Search strategies and results of online databases, including PubMed and Embase.

**Additional file 3.** Detailed individual patient data (IPD) of 15 included studies. Among them, detailed IPD-based survival data of three included studies was reconstructed based on the reported survival curves.

**Additional file 4: Table S1.** Quality assessment of included studies for quantitative synthesis in the systematic review and meta-analysis. **Table S2.** Baseline characteristics of included studies for quantitative synthesis in the systematic review and meta-analysis. **Table S3.** Survival outcomes of included studies for quantitative synthesis in the systematic review and meta-analysis. Majority data is about the HRs (95%CI) of progression-free survival. **Table S4.** Baseline characteristics of the included individual patient data. **Table S5.** Baseline characteristics for patients with different baseline ctDNA statuses (+ vs. -) before and after propensity score matching. **Table S6.** Baseline characteristics for patients with different postoperative ctDNA statuses (+ vs. -) before and after propensity score matching. **Table S7.** Baseline characteristics for patients with different longitudinal ctDNA statuses (+ vs. -) before and after propensity score matching. **Table S8.** Survival outcomes by baseline ctDNA status (+ vs. -) based on reconstructed individual patient data. **Table S9.** Survival outcomes by longitudinal ctDNA status (+ vs. -) based on reconstructed individual patient data. **Table S10.** Negative and positive predictive values of ctDNA at different timepoints by subgroup analysis. **Table S11.** Disease-free survival (DFS) analysis by postoperative ctDNA status and whether ACT was conducted or not: Subgroup analyses by pathological stages and types. **Table S12.** Disease-free survival (DFS) analysis by ctDNA status change from baseline to postoperative timepoints: Subgroup analyses by pathological stages and types.

**Additional file 5: Figure S1.** PRISMA flow of included studies for the systematic review and reconstructed IPD-based meta-analysis. Seven abstract studies were included for qualitative systematic review, 20 studies (14 full-length articles and 6 abstracts) were included for quantitative meta-analysis, and 15 full-length articles were included for IPD-based meta-analysis. **Figure S2.** Summary of major study design models based on the included studies. **Figure S3.** Distribution of propensity scores between ctDNA-positive and -negative groups before and after propensity score matching (PSM) at different timepoints. A, baseline ctDNA; B, postoperative ctDNA; C, longitudinal ctDNA. **Figure S4.** Meta-analysis based on survival outcomes by ctDNA status at different timepoints. A, D, pooled analysis of overall survival (OS) by baseline and postoperative ctDNA statuses respectively; B, E, G: sensitivity analysis of pooled disease-free survival (DFS) outcome by baseline, postoperative, and longitudinal ctDNA statuses respectively; C, F, H: publication bias analysis of pooled DFS outcome by baseline,  postoperative and longitudinal ctDNA statuses respectively. **Figure S5.** A, C: Synthesis of individual patient data-based disease-free survival (DFS) by ctDNA status (+ vs. -) after neoadjuvant therapy (NAT) and adjuvant chemotherapy (ACT) respectively; B, D: pooled meta-analysis of DFS by ctDNA status (+ vs. -) after NAT and ACT respectively. **Figure S6.** Subgroup analysis of individual patient data-based disease-free survival (DFS) by ctDNA status change from baseline to postoperative timepoints. AD, adenocarcinoma; Neg, negative; Pos, positive; SCC, squamous cell carcinoma. **Figure S7.** Subgroup analysis of individual patient data-based disease-free survival (DFS) by postoperative ctDNA status and whether adjuvant chemotherapy (ACT) was conducted or not. AD, adenocarcinoma; Neg, negative; nonACT, without ACT; Pos, positive; SCC, squamous cell carcinoma.

## Data Availability

All study data can be viewed in the supplemental materials.

## Abbreviations

ACT: Adjuvant chemotherapy
AD: Adenocarcinoma of lung
CI: Confidential interval
CPV: Combined predictive value
ctDNA: Circulating tumor DNA
DFS: Disease-free survival
HR: Hazard ratio
ICIs: Immune checkpoint inhibitors
IPD: Individual patient data
NAT: Neoadjuvant therapy
NOS: The Newcastle–Ottawa Quality Assessment Scale
NPV: Negative predictive value
NSCLC: Non-small cell lung cancer
OS: Overall survival
PPV: Positive predictive value
PRISMA: The Preferred Reporting Items for Systematic Reviews and Meta-Analyses
PSM: Propensity score matching
SCC: Squamous cell carcinoma of lung
