# Supplementary figures and images for: Prognostic value of circulating tumor DNA in operable non-small cell lung cancer: a systematic review and reconstructed individual patient-data based meta-analysis
Source: BMC Med. 2023 Nov 27;21:467. doi: 10.1186/s12916-023-03181-2 (PMC10683311; doi:10.1186/s12916-023-03181-2)

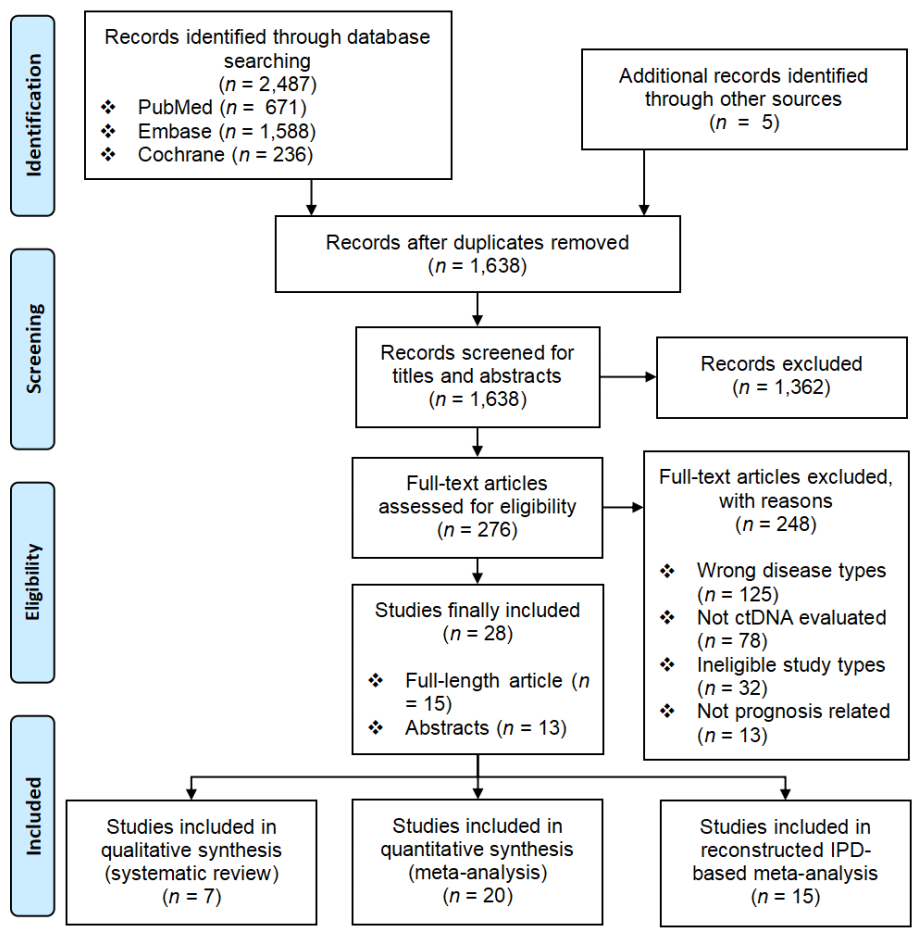

Supplement: Supplementary file 5 — Additional file 5: Figure S1. PRISMA flow of included studies for the systematic review and reconstructed IPD-based meta-analysis. Seven abstract studies were included for qualitative systematic review, 20 studies (14 full-length articles and 6 abstracts) were included for quantitative meta-analysis, and 15 full-length articles were included for IPD-based meta-analysis. Figure S2. Summary of major study design models based on the included studies. Figure S3. Distribution of propensity scores between ctDNA-positive and -negative groups before and after propensity score matching (PSM) at different timepoints. A, baseline ctDNA; B, postoperative ctDNA; C, longitudinal ctDNA. Figure S4. Meta-analysis based on survival outcomes by ctDNA status at different timepoints. A, D, pooled analysis of overall survival (OS) by baseline and postoperative ctDNA statuses respectively; B, E, G: sensitivity analysis of pooled disease-free survival (DFS) outcome by baseline, postoperative, and longitudinal ctDNA statuses respectively; C, F, H: publication bias analysis of pooled DFS outcome by baseline, postoperative and longitudinal ctDNA statuses respectively. Figure S5. A, C: Synthesis of individual patient data-based disease-free survival (DFS) by ctDNA status (+ vs. -) after neoadjuvant therapy (NAT) and adjuvant chemotherapy (ACT) respectively; B, D: pooled meta-analysis of DFS by ctDNA status (+ vs. -) after NAT and ACT respectively. Figure S6. Subgroup analysis of individual patient data-based disease-free survival (DFS) by ctDNA status change from baseline to postoperative timepoints. AD, adenocarcinoma; Neg, negative; Pos, positive; SCC, squamous cell carcinoma. Figure S7. Subgroup analysis of individual patient data-based disease-free survival (DFS) by postoperative ctDNA status and whether adjuvant chemotherapy (ACT) was conducted or not. AD, adenocarcinoma; Neg, negative; nonACT, without ACT; Pos, positive; SCC, squamous cell carcinoma. [file 12916_2023_3181_MOESM5_ESM.zip › Figure S1R2.tif]

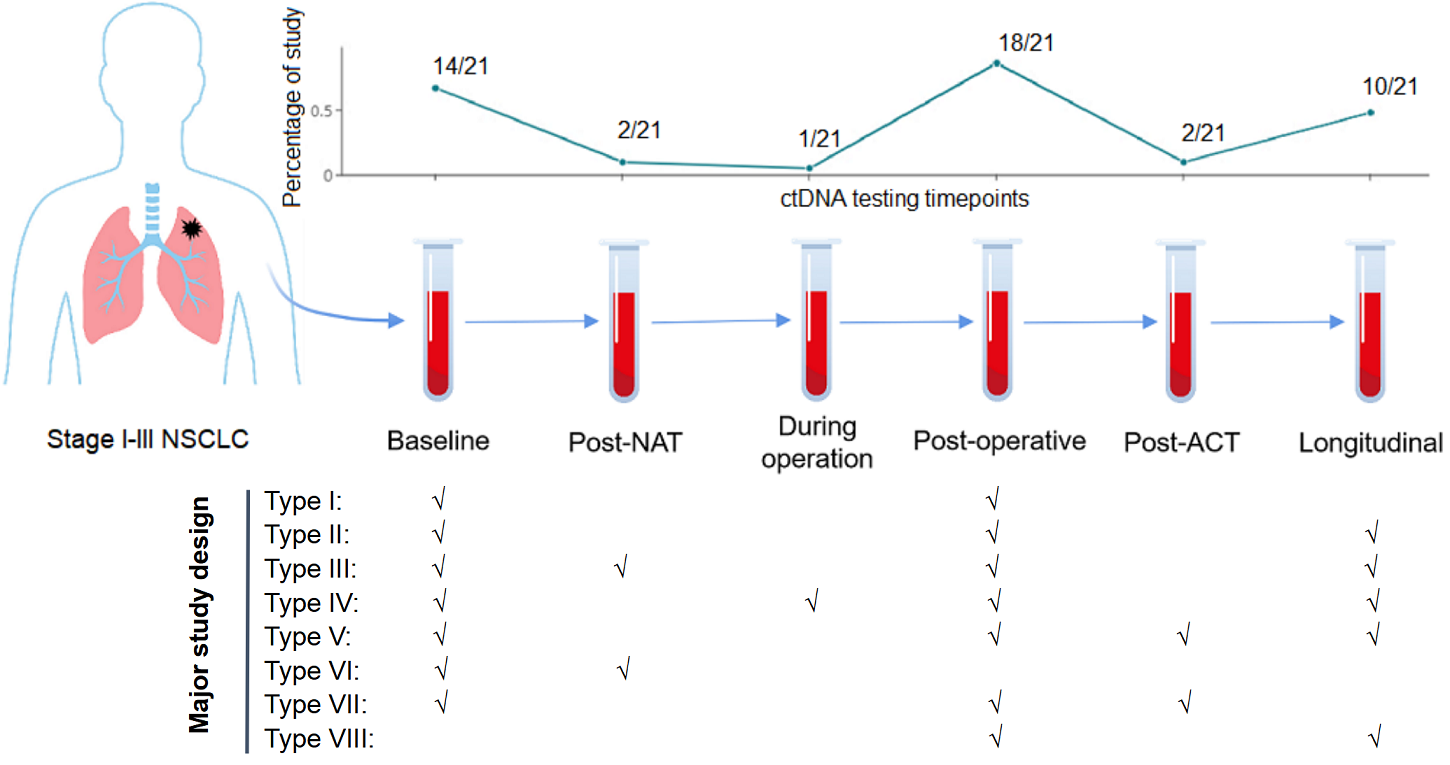

Supplement: Supplementary file 5 — Additional file 5: Figure S1. PRISMA flow of included studies for the systematic review and reconstructed IPD-based meta-analysis. Seven abstract studies were included for qualitative systematic review, 20 studies (14 full-length articles and 6 abstracts) were included for quantitative meta-analysis, and 15 full-length articles were included for IPD-based meta-analysis. Figure S2. Summary of major study design models based on the included studies. Figure S3. Distribution of propensity scores between ctDNA-positive and -negative groups before and after propensity score matching (PSM) at different timepoints. A, baseline ctDNA; B, postoperative ctDNA; C, longitudinal ctDNA. Figure S4. Meta-analysis based on survival outcomes by ctDNA status at different timepoints. A, D, pooled analysis of overall survival (OS) by baseline and postoperative ctDNA statuses respectively; B, E, G: sensitivity analysis of pooled disease-free survival (DFS) outcome by baseline, postoperative, and longitudinal ctDNA statuses respectively; C, F, H: publication bias analysis of pooled DFS outcome by baseline, postoperative and longitudinal ctDNA statuses respectively. Figure S5. A, C: Synthesis of individual patient data-based disease-free survival (DFS) by ctDNA status (+ vs. -) after neoadjuvant therapy (NAT) and adjuvant chemotherapy (ACT) respectively; B, D: pooled meta-analysis of DFS by ctDNA status (+ vs. -) after NAT and ACT respectively. Figure S6. Subgroup analysis of individual patient data-based disease-free survival (DFS) by ctDNA status change from baseline to postoperative timepoints. AD, adenocarcinoma; Neg, negative; Pos, positive; SCC, squamous cell carcinoma. Figure S7. Subgroup analysis of individual patient data-based disease-free survival (DFS) by postoperative ctDNA status and whether adjuvant chemotherapy (ACT) was conducted or not. AD, adenocarcinoma; Neg, negative; nonACT, without ACT; Pos, positive; SCC, squamous cell carcinoma. [file 12916_2023_3181_MOESM5_ESM.zip › Figure S2R2.tif]

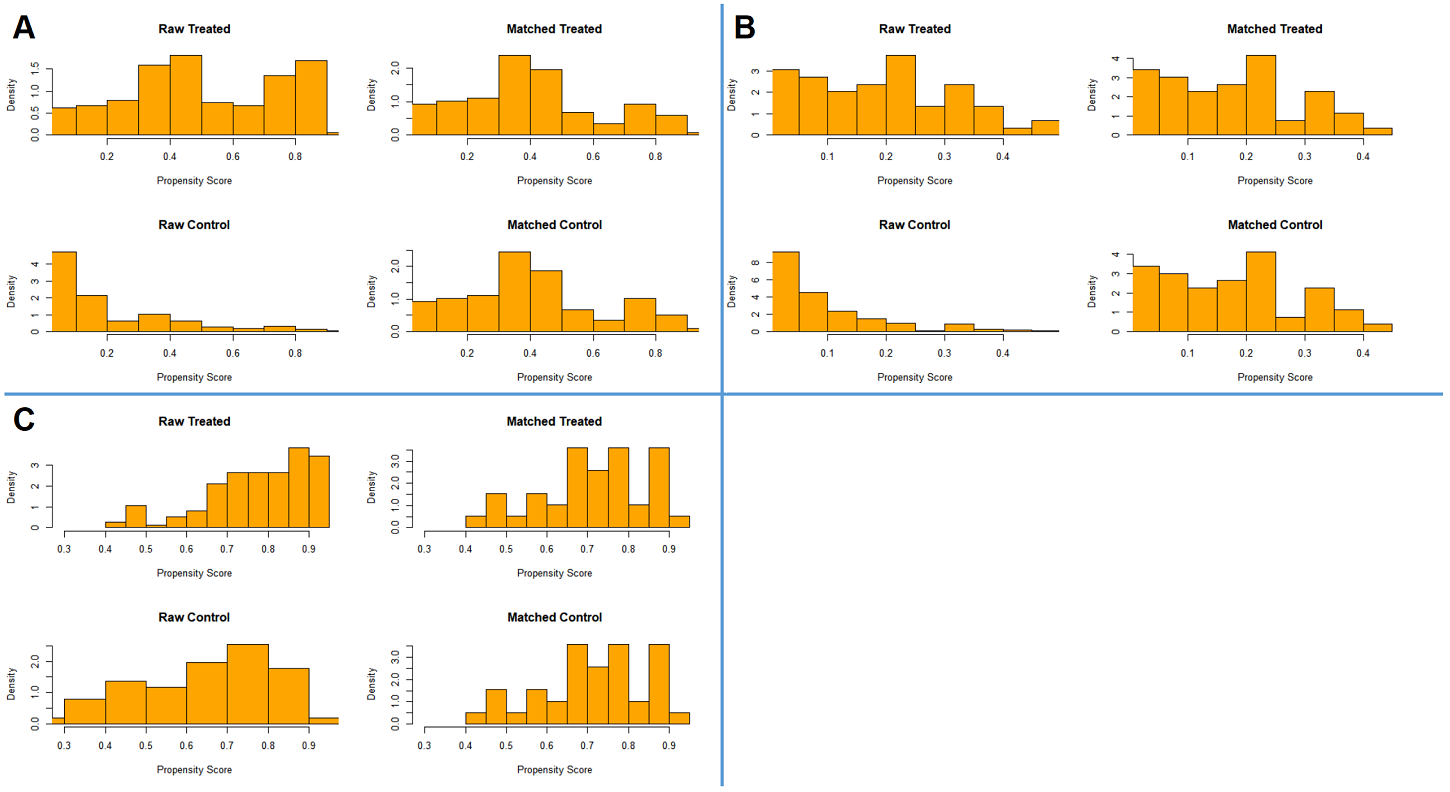

Supplement: Supplementary file 5 — Additional file 5: Figure S1. PRISMA flow of included studies for the systematic review and reconstructed IPD-based meta-analysis. Seven abstract studies were included for qualitative systematic review, 20 studies (14 full-length articles and 6 abstracts) were included for quantitative meta-analysis, and 15 full-length articles were included for IPD-based meta-analysis. Figure S2. Summary of major study design models based on the included studies. Figure S3. Distribution of propensity scores between ctDNA-positive and -negative groups before and after propensity score matching (PSM) at different timepoints. A, baseline ctDNA; B, postoperative ctDNA; C, longitudinal ctDNA. Figure S4. Meta-analysis based on survival outcomes by ctDNA status at different timepoints. A, D, pooled analysis of overall survival (OS) by baseline and postoperative ctDNA statuses respectively; B, E, G: sensitivity analysis of pooled disease-free survival (DFS) outcome by baseline, postoperative, and longitudinal ctDNA statuses respectively; C, F, H: publication bias analysis of pooled DFS outcome by baseline, postoperative and longitudinal ctDNA statuses respectively. Figure S5. A, C: Synthesis of individual patient data-based disease-free survival (DFS) by ctDNA status (+ vs. -) after neoadjuvant therapy (NAT) and adjuvant chemotherapy (ACT) respectively; B, D: pooled meta-analysis of DFS by ctDNA status (+ vs. -) after NAT and ACT respectively. Figure S6. Subgroup analysis of individual patient data-based disease-free survival (DFS) by ctDNA status change from baseline to postoperative timepoints. AD, adenocarcinoma; Neg, negative; Pos, positive; SCC, squamous cell carcinoma. Figure S7. Subgroup analysis of individual patient data-based disease-free survival (DFS) by postoperative ctDNA status and whether adjuvant chemotherapy (ACT) was conducted or not. AD, adenocarcinoma; Neg, negative; nonACT, without ACT; Pos, positive; SCC, squamous cell carcinoma. [file 12916_2023_3181_MOESM5_ESM.zip › Figure S3R2.tif]

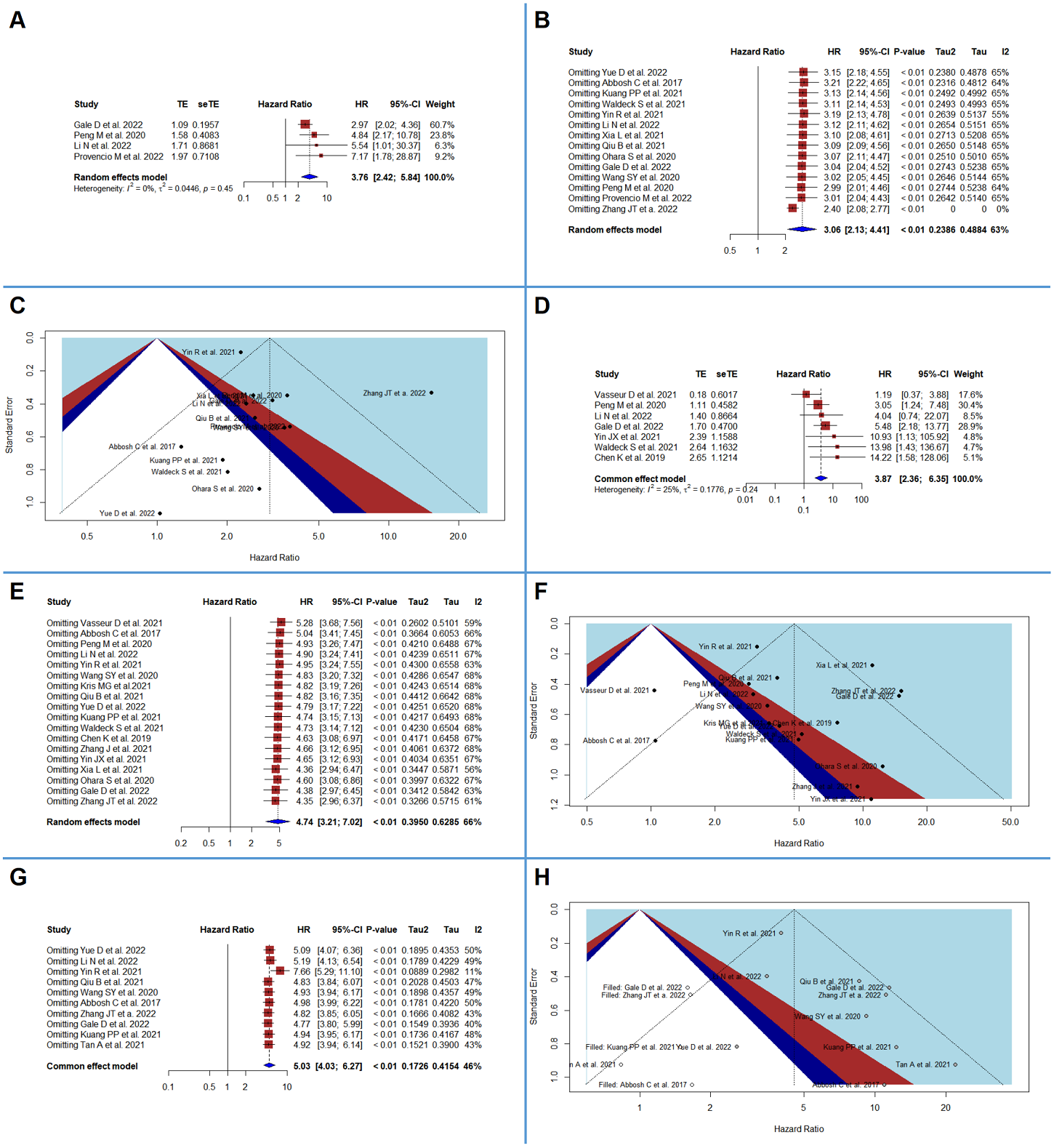

Supplement: Supplementary file 5 — Additional file 5: Figure S1. PRISMA flow of included studies for the systematic review and reconstructed IPD-based meta-analysis. Seven abstract studies were included for qualitative systematic review, 20 studies (14 full-length articles and 6 abstracts) were included for quantitative meta-analysis, and 15 full-length articles were included for IPD-based meta-analysis. Figure S2. Summary of major study design models based on the included studies. Figure S3. Distribution of propensity scores between ctDNA-positive and -negative groups before and after propensity score matching (PSM) at different timepoints. A, baseline ctDNA; B, postoperative ctDNA; C, longitudinal ctDNA. Figure S4. Meta-analysis based on survival outcomes by ctDNA status at different timepoints. A, D, pooled analysis of overall survival (OS) by baseline and postoperative ctDNA statuses respectively; B, E, G: sensitivity analysis of pooled disease-free survival (DFS) outcome by baseline, postoperative, and longitudinal ctDNA statuses respectively; C, F, H: publication bias analysis of pooled DFS outcome by baseline, postoperative and longitudinal ctDNA statuses respectively. Figure S5. A, C: Synthesis of individual patient data-based disease-free survival (DFS) by ctDNA status (+ vs. -) after neoadjuvant therapy (NAT) and adjuvant chemotherapy (ACT) respectively; B, D: pooled meta-analysis of DFS by ctDNA status (+ vs. -) after NAT and ACT respectively. Figure S6. Subgroup analysis of individual patient data-based disease-free survival (DFS) by ctDNA status change from baseline to postoperative timepoints. AD, adenocarcinoma; Neg, negative; Pos, positive; SCC, squamous cell carcinoma. Figure S7. Subgroup analysis of individual patient data-based disease-free survival (DFS) by postoperative ctDNA status and whether adjuvant chemotherapy (ACT) was conducted or not. AD, adenocarcinoma; Neg, negative; nonACT, without ACT; Pos, positive; SCC, squamous cell carcinoma. [file 12916_2023_3181_MOESM5_ESM.zip › Figure S4R2.tif]

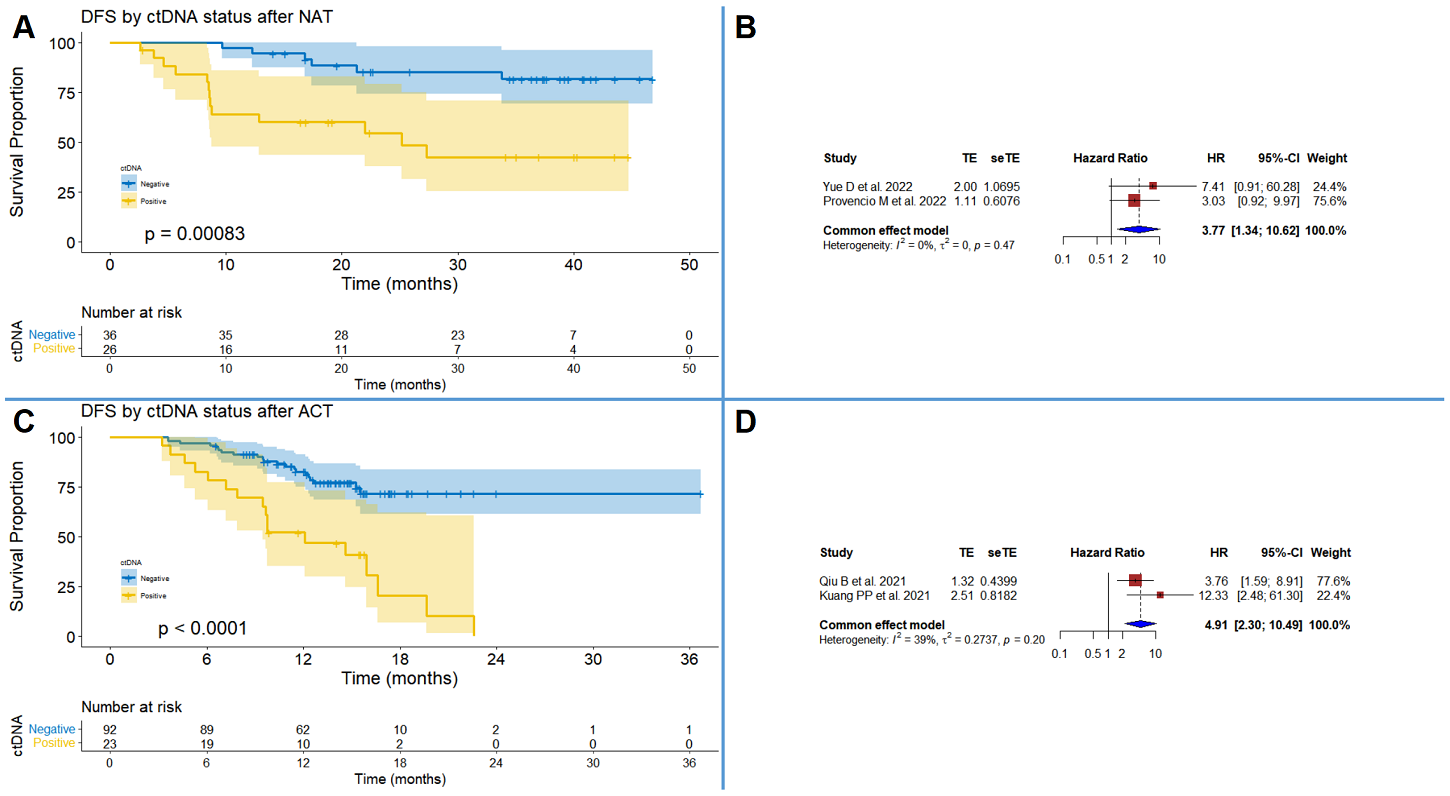

Supplement: Supplementary file 5 — Additional file 5: Figure S1. PRISMA flow of included studies for the systematic review and reconstructed IPD-based meta-analysis. Seven abstract studies were included for qualitative systematic review, 20 studies (14 full-length articles and 6 abstracts) were included for quantitative meta-analysis, and 15 full-length articles were included for IPD-based meta-analysis. Figure S2. Summary of major study design models based on the included studies. Figure S3. Distribution of propensity scores between ctDNA-positive and -negative groups before and after propensity score matching (PSM) at different timepoints. A, baseline ctDNA; B, postoperative ctDNA; C, longitudinal ctDNA. Figure S4. Meta-analysis based on survival outcomes by ctDNA status at different timepoints. A, D, pooled analysis of overall survival (OS) by baseline and postoperative ctDNA statuses respectively; B, E, G: sensitivity analysis of pooled disease-free survival (DFS) outcome by baseline, postoperative, and longitudinal ctDNA statuses respectively; C, F, H: publication bias analysis of pooled DFS outcome by baseline, postoperative and longitudinal ctDNA statuses respectively. Figure S5. A, C: Synthesis of individual patient data-based disease-free survival (DFS) by ctDNA status (+ vs. -) after neoadjuvant therapy (NAT) and adjuvant chemotherapy (ACT) respectively; B, D: pooled meta-analysis of DFS by ctDNA status (+ vs. -) after NAT and ACT respectively. Figure S6. Subgroup analysis of individual patient data-based disease-free survival (DFS) by ctDNA status change from baseline to postoperative timepoints. AD, adenocarcinoma; Neg, negative; Pos, positive; SCC, squamous cell carcinoma. Figure S7. Subgroup analysis of individual patient data-based disease-free survival (DFS) by postoperative ctDNA status and whether adjuvant chemotherapy (ACT) was conducted or not. AD, adenocarcinoma; Neg, negative; nonACT, without ACT; Pos, positive; SCC, squamous cell carcinoma. [file 12916_2023_3181_MOESM5_ESM.zip › Figure S5R2.tif]

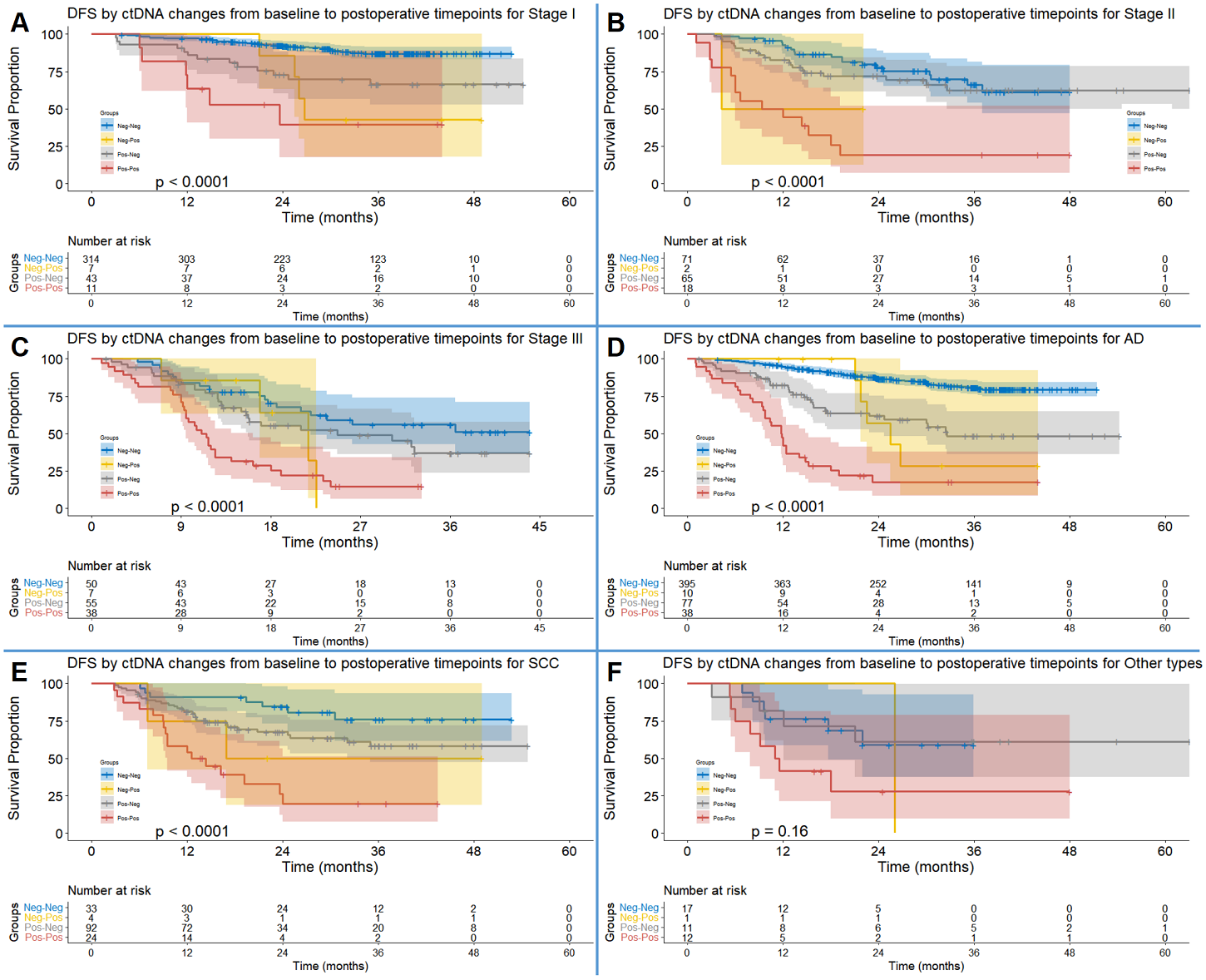

Supplement: Supplementary file 5 — Additional file 5: Figure S1. PRISMA flow of included studies for the systematic review and reconstructed IPD-based meta-analysis. Seven abstract studies were included for qualitative systematic review, 20 studies (14 full-length articles and 6 abstracts) were included for quantitative meta-analysis, and 15 full-length articles were included for IPD-based meta-analysis. Figure S2. Summary of major study design models based on the included studies. Figure S3. Distribution of propensity scores between ctDNA-positive and -negative groups before and after propensity score matching (PSM) at different timepoints. A, baseline ctDNA; B, postoperative ctDNA; C, longitudinal ctDNA. Figure S4. Meta-analysis based on survival outcomes by ctDNA status at different timepoints. A, D, pooled analysis of overall survival (OS) by baseline and postoperative ctDNA statuses respectively; B, E, G: sensitivity analysis of pooled disease-free survival (DFS) outcome by baseline, postoperative, and longitudinal ctDNA statuses respectively; C, F, H: publication bias analysis of pooled DFS outcome by baseline, postoperative and longitudinal ctDNA statuses respectively. Figure S5. A, C: Synthesis of individual patient data-based disease-free survival (DFS) by ctDNA status (+ vs. -) after neoadjuvant therapy (NAT) and adjuvant chemotherapy (ACT) respectively; B, D: pooled meta-analysis of DFS by ctDNA status (+ vs. -) after NAT and ACT respectively. Figure S6. Subgroup analysis of individual patient data-based disease-free survival (DFS) by ctDNA status change from baseline to postoperative timepoints. AD, adenocarcinoma; Neg, negative; Pos, positive; SCC, squamous cell carcinoma. Figure S7. Subgroup analysis of individual patient data-based disease-free survival (DFS) by postoperative ctDNA status and whether adjuvant chemotherapy (ACT) was conducted or not. AD, adenocarcinoma; Neg, negative; nonACT, without ACT; Pos, positive; SCC, squamous cell carcinoma. [file 12916_2023_3181_MOESM5_ESM.zip › Figure S6R2.tif]

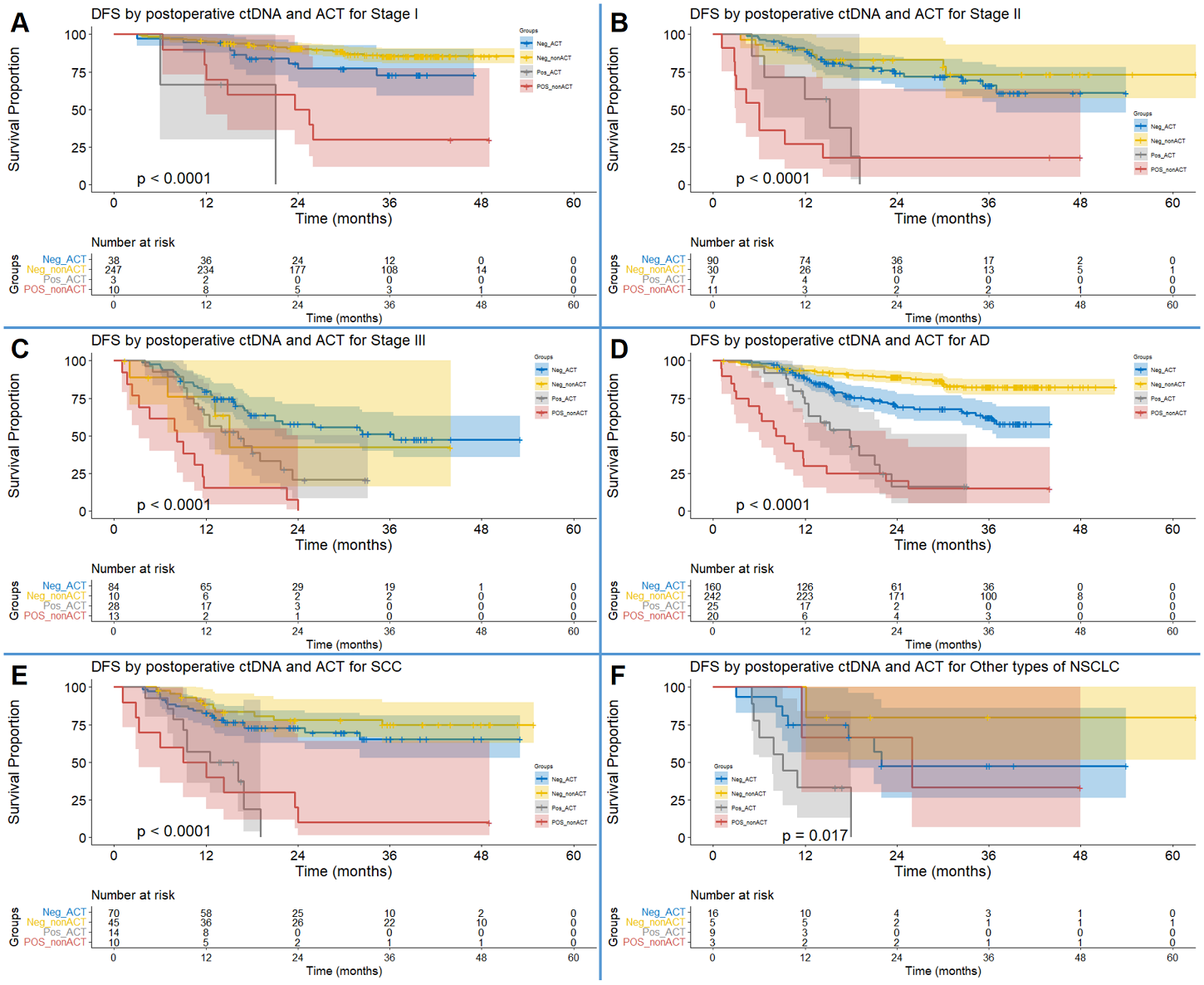

Supplement: Supplementary file 5 — Additional file 5: Figure S1. PRISMA flow of included studies for the systematic review and reconstructed IPD-based meta-analysis. Seven abstract studies were included for qualitative systematic review, 20 studies (14 full-length articles and 6 abstracts) were included for quantitative meta-analysis, and 15 full-length articles were included for IPD-based meta-analysis. Figure S2. Summary of major study design models based on the included studies. Figure S3. Distribution of propensity scores between ctDNA-positive and -negative groups before and after propensity score matching (PSM) at different timepoints. A, baseline ctDNA; B, postoperative ctDNA; C, longitudinal ctDNA. Figure S4. Meta-analysis based on survival outcomes by ctDNA status at different timepoints. A, D, pooled analysis of overall survival (OS) by baseline and postoperative ctDNA statuses respectively; B, E, G: sensitivity analysis of pooled disease-free survival (DFS) outcome by baseline, postoperative, and longitudinal ctDNA statuses respectively; C, F, H: publication bias analysis of pooled DFS outcome by baseline, postoperative and longitudinal ctDNA statuses respectively. Figure S5. A, C: Synthesis of individual patient data-based disease-free survival (DFS) by ctDNA status (+ vs. -) after neoadjuvant therapy (NAT) and adjuvant chemotherapy (ACT) respectively; B, D: pooled meta-analysis of DFS by ctDNA status (+ vs. -) after NAT and ACT respectively. Figure S6. Subgroup analysis of individual patient data-based disease-free survival (DFS) by ctDNA status change from baseline to postoperative timepoints. AD, adenocarcinoma; Neg, negative; Pos, positive; SCC, squamous cell carcinoma. Figure S7. Subgroup analysis of individual patient data-based disease-free survival (DFS) by postoperative ctDNA status and whether adjuvant chemotherapy (ACT) was conducted or not. AD, adenocarcinoma; Neg, negative; nonACT, without ACT; Pos, positive; SCC, squamous cell carcinoma. [file 12916_2023_3181_MOESM5_ESM.zip › Figure S7R2.tif]
